# A safety risk assessment checklist for personalized exercise as early supportive care in breast cancer patients undergoing chemotherapy: a modified Delphi consensus study

**DOI:** 10.1186/s12904-026-02083-3

**Published:** 2026-04-01

**Authors:** Xinbo Du, Huijie Wei, Wubin He, Ying Song

**Affiliations:** 1 Department of Urolithiasis Ward, First Affiliated Hospital of Jinzhou Medical University, Jinzhou, China; 2 School of Nursing, Jinzhou Medical University, Jinzhou, China; 3 Key Laboratory of Surgery of Liaoning Province, First Affiliated Hospital of Jinzhou Medical University, Jinzhou, China; 4 Cardiovascular Laboratory, First Affiliated Hospital of Jinzhou Medical University, Jinzhou, China

**Keywords:** Breast cancer, Supportive care, Exercise, Risk assessment, Symptom burden, Quality of life, Oncology nursing, Delphi technique

## Abstract

**Background:**

Exercise is an effective non-pharmacological intervention to alleviate the high symptom burden and mitigate declines in physical function and quality of life for breast cancer patients undergoing chemotherapy. Its integration into routine supportive care is challenged by clinicians’ uncertainty in assessing dynamic, treatment-specific risks. This study aimed to develop a consensus-based safety assessment tool to facilitate the safe implementation of exercise as part of individualized supportive care.

**Methods:**

A multi-method, two-phase study was conducted (scoping review followed by Delphi consensus, rather than a mixed-methods design, as the phases were conducted sequentially without formal integration of qualitative and quantitative data). First, a preliminary checklist was drafted via a structured scoping review of guidelines, literature, and existing tools (2012–2025). Second, a three-round modified online Delphi consensus process was undertaken with a multidisciplinary panel of 20 experts (clinical oncology, oncology nursing, exercise physiology, physical therapy). Consensus was defined a priori as ≥ 80% agreement on relevance, with a median ≥ 4.0 on a 5-point Likert scale. Items were revised iteratively based on quantitative ratings and qualitative feedback.

**Results:**

All panelists (100%) completed all rounds. Final consensus was achieved on a 25-item checklist, structured into four domains: (1) Medical & Treatment-Related Factors (e.g., neutropenia, timing post-infusion), (2) Symptom Burden (e.g., severe fatigue, pain), (3) Functional & Mobility Considerations (e.g., neuropathy, balance issues), and (4) Patient-Specific Context (e.g., comorbidities, anxiety). A novel risk stratification guide translates checklist findings into a three-tiered decision pathway: “Red Light” (defer exercise, consult oncology/supportive care team), “Yellow Light” (proceed with tailored modifications), and “Green Light” (proceed with personalized prescription). This provides a clear framework for clinical decision-making.

**Conclusions:**

This Delphi study establishes a multidisciplinary expert consensus on a pragmatic safety assessment checklist and decision pathway. The tool is designed to empower oncology and supportive care clinicians, particularly nurses, in integrating personalized exercise into symptom management plans safely. By standardizing risk evaluation, it addresses a key barrier to implementing exercise as supportive care and may enhance patient-clinician communication. The present study establishes content validity—the essential first step in tool development. Following the MRC framework for complex interventions, the next phases will involve: (1) a pilot feasibility study to establish administration protocols, assess acceptability, and refine items; (2) a prospective implementation study to evaluate impact on clinical decision-making and patient outcomes. Future research must also validate its feasibility and impact on symptom outcomes and quality of life, and explore its adaptation for patients with advanced disease, with explicit involvement of palliative care specialists.

**Supplementary Information:**

The online version contains supplementary material available at 10.1186/s12904-026-02083-3.

## Introduction

Breast cancer remains the most prevalent malignancy among women globally, with chemotherapy constituting a cornerstone of treatment for many patients [1]. While effective in improving survival, chemotherapy is associated with a significant symptom burden—including debilitating fatigue, pain, peripheral neuropathy, and declines in physical function—that profoundly impairs patients’ quality of life (QoL) and functional independence [2]. In this context, exercise has emerged as a fundamental, evidence-based component of supportive care. Substantial evidence demonstrates that structured exercise during chemotherapy can mitigate these treatment-related side effects, enhance cardiopulmonary fitness, preserve musculoskeletal integrity, and improve psychological well-being [3–5].

In this manuscript, we use the term “exercise” to refer to planned, structured, and repetitive physical activity undertaken with the goal of improving or maintaining physical fitness, function, or well-being [adapted from [6]. This includes activities ranging from low-intensity walking programs to moderate-intensity aerobic and resistance training, but excludes unstructured activities of daily living. Importantly, the intensity and mode of exercise are expected to be adapted based on the patient’s current clinical status—a principle operationalized by the checklist’s “Yellow Light” pathway.

Consequently, leading oncology organizations now advocate for the integration of exercise into standard cancer care, recommending that patients avoid inactivity and engage in regular physical activity [7, 8]. This recommendation aligns with the core principles of early palliative and supportive care, which aims to prevent and relieve suffering and to improve QoL for patients with serious illness, regardless of prognosis [9]. For oncology and supportive care clinicians at the point of care, particularly nurses, translating these guidelines into safe, personalized, and symptom-responsive exercise plans for patients undergoing active chemotherapy presents a complex challenge. This population exhibits marked heterogeneity in treatment protocols, dynamic symptom trajectories, baseline functional status, and comorbidities, all of which modulate individual tolerance and risk associated with physical activity [10, 11]. Ensuring safety is paramount, as inappropriate exercise prescription could exacerbate severe fatigue, increase injury risk in patients with chemotherapy-induced neuropathy, or stress cardiovascular systems compromised by specific agents [12]. This uncertainty represents a critical barrier to implementing exercise as routine supportive care, potentially leading to the underuse of a beneficial non-pharmacological intervention [13, 14].

In current nursing and supportive care practice, safety assessment for exercise often relies on ad hoc screening or individual judgement, approaches that lack standardization and can perpetuate clinical hesitancy [14]. While general pre-participation screening tools exist, they lack specificity for the unique, dynamic, and treatment-contingent risks associated with cancer therapy, such as fluctuating blood counts, the timing relative to infusion, and the management of active symptoms [15]. A standardized, pragmatic tool—designed as a concise, bedside checklist that integrates these oncology-specific factors and provides a clear clinical decision pathway—is notably absent from the supportive care toolkit [16]. This gap leaves clinicians without a consistent framework to support critical safety judgements, tailor interventions to the individual’s symptom profile, and facilitate patient education regarding safe exercise participation [17]. Such a tool is critically needed to bridge the well-documented gap between guideline recommendations and their implementation in practice, thereby enabling the safer integration of exercise into personalized supportive care plans [18].

While the ACSM’s FITT-VP framework (Frequency, Intensity, Time, Type, Volume, Progression) provides the foundation for exercise prescription in general populations [16], its application in oncology requires adaptation to account for treatment-specific risks and symptom fluctuations [10]. The present checklist is designed as a pre-prescription safety screening tool that operationalizes the ‘screening’ and ‘risk stratification’ steps preceding the application of FITT-VP principles in the oncology setting. For breast cancer survivors, specific adaptations to ACSM intensity prescriptions may be warranted, as demonstrated by Sweegers et al. [19].

Breast cancer was selected as the index condition for this study for several reasons. First, breast cancer is the most prevalent malignancy among women globally [1], with a large and diverse patient population undergoing chemotherapy. Second, the evidence base for exercise during breast cancer treatment is particularly well-established, with multiple randomized controlled trials and meta-analyses demonstrating benefits for symptom management, physical function, and quality of life [3–5]. Third, breast cancer chemotherapy regimens are heterogeneous, involving different agents, schedules, and side effect profiles, which necessitates individualized safety assessment. Finally, breast cancer survivors face specific treatment-related sequelae—such as upper extremity morbidity, cardiotoxicity risk, and persistent fatigue—that require careful consideration in exercise prescription [10]. These characteristics make breast cancer an ideal model for developing a safety assessment tool that may later be adapted for other cancer populations.

Therefore, to address this gap and empower frontline clinicians in delivering holistic supportive care, this study aimed to develop and achieve expert consensus on a pragmatic, domain-based Safety Risk Assessment Checklist and clinical decision pathway specifically designed for breast cancer patients undergoing chemotherapy. The goal was to provide a directly applicable tool to facilitate the safe integration of personalized exercise into symptom management and supportive care for this population.

## Methods

### Study design and overview

**Fig. 1:**
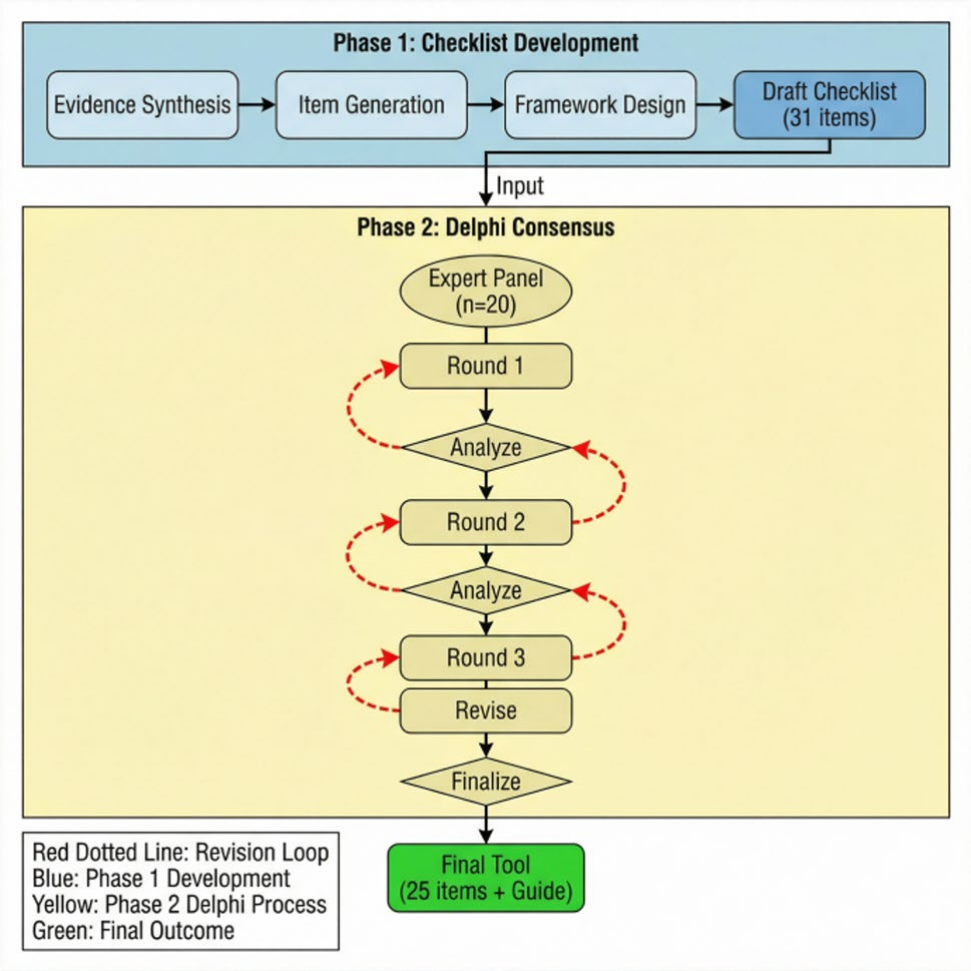
Study design flowchart for the development of the safety risk assessment checklist

This study employed a sequential two-phase, multi-method design (Fig. 1). Phase 1 involved the development of a preliminary checklist draft through a structured scoping review of evidence and guidelines, focusing on factors pertinent to safe exercise within the supportive care context. Phase 2 utilized a formal, three-round modified Delphi technique to refine the draft and establish multidisciplinary expert consensus. This constitutes a multi-method rather than a mixed-methods design, as the phases were conducted sequentially without formal integration of qualitative and quantitative data. The study was conducted between March 2025 and November 2025. The protocol received ethical approval from an authorized Institutional Review Board. As the Delphi process involved collecting professional opinions for consensus development without patient data or interventions, the requirement for written informed consent was waived. All methods were performed in accordance with the Declaration of Helsinki.

### Phase 1: development of the preliminary checklist draft

#### Evidence synthesis and item generation

We generated the initial list of items by conducting a structured scoping review, guided by the Arksey & O’Malley framework [20]. The process systematically integrated evidence from three complementary sources to capture factors influencing exercise safety within the supportive care context for patients undergoing active treatment:

##### Published clinical practice guidelines

We reviewed major international oncology, supportive care, and exercise guidelines (e.g. [7, 8]), to extract established safety considerations.

##### Existing risk screening tools

We examined published tools for pre-exercise screening in general and oncology settings (e.g. [16]), , analyzing and contextually adapting their items.

##### Empirical research literature

A targeted search of PubMed and CINAHL databases (covering 2012–2025) was performed. PubMed and CINAHL were selected as the primary databases due to their comprehensive coverage of biomedical literature and nursing/allied health literature, respectively, ensuring capture of both clinical and supportive care perspectives. The time frame of 2012–2025 was chosen to capture literature published since the 2012 ACSM roundtable on cancer survivorship, which marked a pivotal moment in exercise oncology guideline development. The search strategy combined keywords and MeSH terms related to “breast neoplasms,” “drug therapy,” “exercise,” “safety,” “adverse events,” and “symptoms.” The full search strings for both databases are provided in Supplementary File 1. This aimed to identify patient-, treatment-, and symptom-specific factors influencing exercise tolerance and safety, with a focus on modifiable risks relevant to supportive care planning [15].

**Fig. 2:**
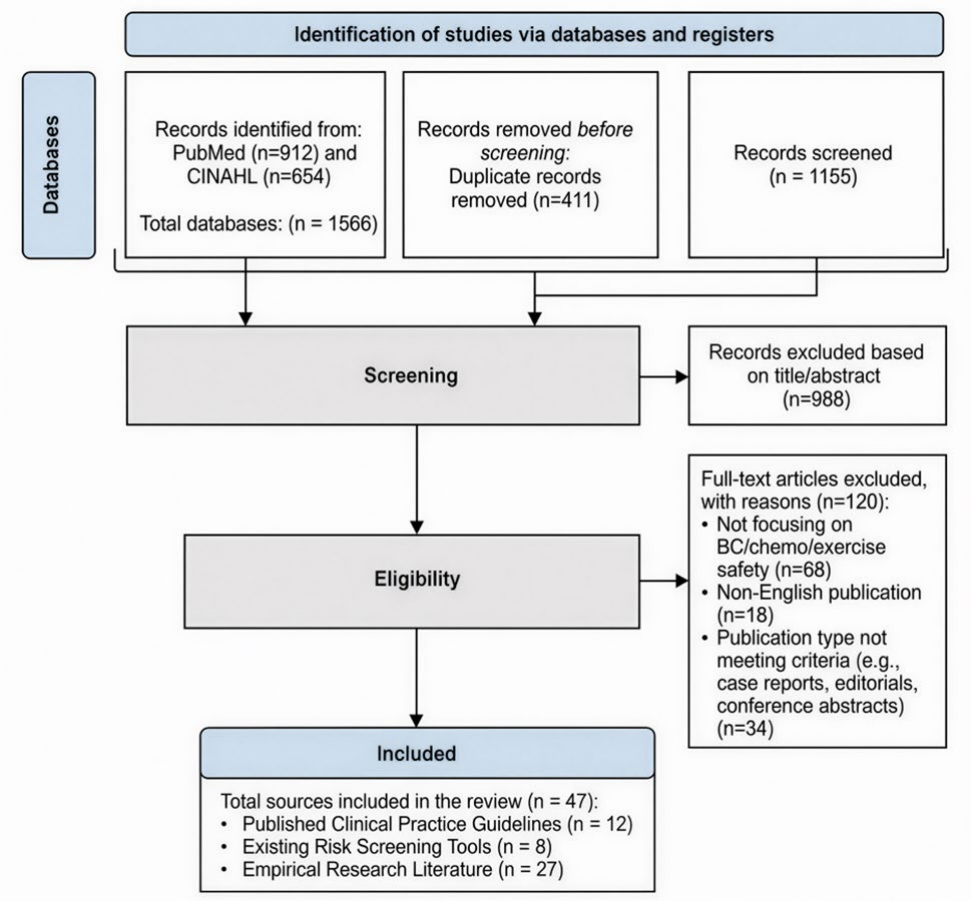
Preferred reporting items for systematic reviews and meta-analyses extension for scoping reviews (PRISMA-ScR) flow diagram of the study selection process

Two authors (X.D. and H.W.) independently screened titles, abstracts, and full texts using predefined inclusion/exclusion criteria, with discrepancies resolved through consensus or by a third reviewer. Inclusion criteria were: (1) English language; (2) published 2012–2025; (3) addressed breast cancer, chemotherapy, and exercise/physical activity; (4) reported on safety considerations, adverse events, or contraindications. Exclusion criteria were: (1) case reports, editorials, conference abstracts; (2) pediatric populations; (3) non-English language. The selection process followed best practices for scoping reviews, with the goal of comprehensively mapping key safety factors rather than appraising individual study quality [20]. A PRISMA-ScR flow diagram documenting the screening process is provided in Fig. 2. Data extraction was performed independently by two authors using a standardized extraction form (Supplementary File 1), capturing: author/year, study type, population, safety factors identified, and relevant recommendations.

The scoping review identified 47 eligible sources: 12 clinical practice guidelines, 8 existing screening tools, and 27 empirical studies. From these sources, an initial pool of 68 potential safety factors was extracted, which were consolidated into a 31-item preliminary checklist after removing duplicates and merging related concepts.

#### Initial framework categorisation

Item derivation followed a structured process: (1) two authors independently extracted potential safety factors from included sources; (2) extracted factors were categorized into provisional domains through iterative discussion; (3) a consensus meeting was held to refine wording and resolve discrepancies. For example, the item “within 48 hours before or after chemotherapy infusion” was derived from expert clinical input regarding acute symptom peaks and infusion reactions, supplemented by limited evidence on optimal timing for exercise relative to treatment. We acknowledge that this threshold lacks strong empirical support and requires validation in future studies. Preliminary items were organized into four provisional domains informed by clinical logic and the synthesized evidence: (1) Medical & Treatment Status, (2) Symptom Burden, (3) Functional & Mobility Limitations, and (4) Patient Context & Readiness. This draft served as the foundation for the Delphi Round 1 survey. The initial draft and full search strategy are documented in Supplementary File 1.

### Phase 2: modified delphi consensus process

#### Expert panel recruitment

A purposive sample of 20 multidisciplinary experts was recruited to ensure representation of key stakeholder groups involved in oncology supportive care and rehabilitation. Experts were identified through: (1) authorship of relevant guidelines/publications in exercise oncology; (2) leadership roles in professional organizations (e.g., ACSM, ASCO, MASCC); (3) snowball sampling from initial contacts. Invitations were sent via email to 32 potential experts; 20 agreed to participate and completed all rounds (62.5% acceptance rate). Inclusion criteria required: (a) ≥ 5 years of relevant professional experience in oncology care, exercise oncology, or cancer rehabilitation; (b) current active practice or research; and (c) commitment to complete all Delphi rounds. The panel comprised clinical oncologists (*n* = 5), oncology nurse specialists (*n* = 5), exercise physiologists/scientists (*n* = 5), and physical therapists/rehabilitation specialists (*n* = 5). All panelists had direct experience managing the symptom burden and functional needs of breast cancer patients. Panelists were recruited internationally: 12 from China, 5 from Europe (Germany, UK, Netherlands), and 3 from North America (USA, Canada). Panelist characteristics are detailed in Supplementary File 2.

#### Delphi procedure

A three-round online Delphi process was administered via a secure survey platform (Qualtrics).

Round 1: Experts rated each preliminary item on a 5-point Likert scale for Relevance (to safe exercise prescription in this population) and Clarity, with open-ended fields for suggestions.

Round 2: Experts re-rated a revised checklist, which incorporated Round 1 feedback. They received a summary of the group’s median ratings and their own previous scores.

Round 3: Experts provided final ratings on items that did not achieve consensus in Round 2, presented with revised wording.

The full questionnaires for all three rounds are provided in Supplementary File 1.

#### Data analysis and consensus definition

Descriptive statistics (median, interquartile range [IQR], percentage agreement) were calculated for each item. Pre-defined consensus criteria were informed by established methodological guidance [19–21]:

##### Consensus IN

Median relevance ≥ 4.0 AND IQR ≤ 1.0 AND ≥ 80% of ratings ≥ 4 (indicating strong agreement on inclusion).

##### Consensus OUT

Median relevance ≤ 2.0 AND ≥ 70% of ratings ≤ 2 (indicating strong agreement on exclusion).

##### No consensus

Items meeting neither criterion (indicating disagreement or uncertainty requiring further refinement).

This three-tiered classification allows for clear decision-making: items achieving Consensus IN were retained; items achieving Consensus OUT were removed; items with No Consensus but median ≥ 3.0 were revised based on qualitative feedback and re-rated in subsequent rounds [21]. Analyses were performed using SPSS Statistics (Version 26.0). A complete record of ratings and item evolution across rounds is provided in Supplementary Table S1.

## Results

### Expert panel characteristics

All 20 invited experts agreed to participate and completed all three Delphi rounds (100% response rate). The multidisciplinary panel comprised clinical oncologists (*n* = 5), oncology nurse specialists (*n* = 5), exercise physiologists/scientists (*n* = 5), and physical therapists/rehabilitation specialists (*n* = 5). Participants had a mean professional experience of 14.7 years (SD = 5.2) in oncology care, rehabilitation, or related research. All panelists were actively engaged in managing the symptom burden and functional needs of breast cancer patients. Detailed demographic and professional backgrounds, including individual expert profiles and organizational affiliations, are provided in Supplementary File 2 (Table S2.1).

### Delphi process outcomes

**Fig. 3:**
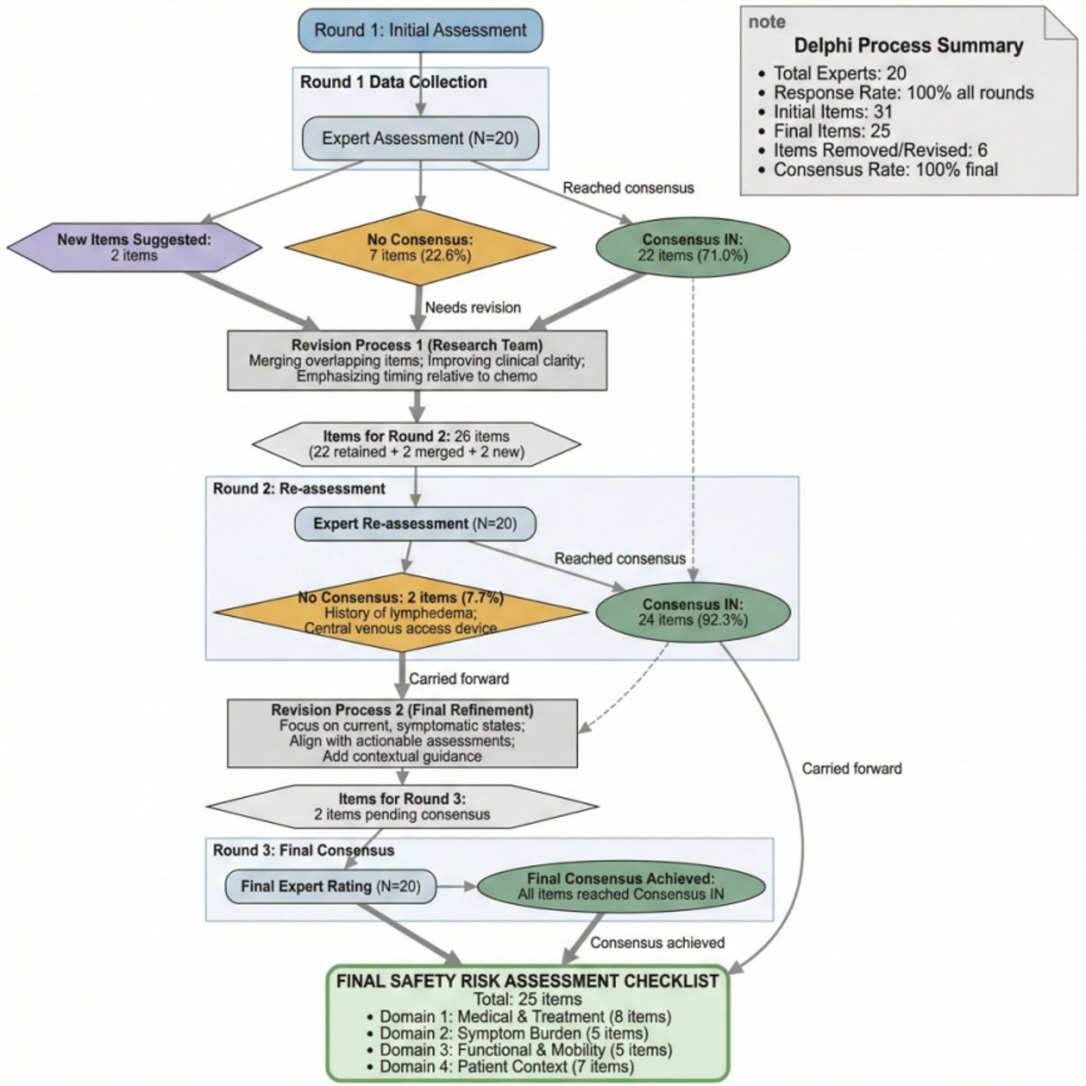
Flow diagram of checklist items through the Delphi consensus rounds

The three-round modified Delphi process successfully refined the checklist with strong consensus achieved on all final items. The flow of items through the rounds is summarized in Fig. 3.

Round 1:The preliminary 31-item checklist was assessed. Twenty-two items achieved immediate “Consensus IN.” Qualitative feedback was instrumental in refining clinical specificity, with experts emphasizing the need to assess dynamic symptom status and treatment timing—key considerations for supportive care planning.

Round 2: A revised 26-item checklist was rated. Twenty-four items achieved “Consensus IN.” Two items required further revision based on expert deliberation, which centered on ensuring the tool promoted safe adaptation rather than unwarranted exclusion for conditions like lymphedema.

Round 3:The two revised items, incorporating nuanced feedback to focus on active, symptomatic states, achieved “Consensus IN.”

A complete record of statistical ratings and the evolution of all items across rounds is available in Supplementary Table S1.

### The final safety risk assessment checklist

The expert panel achieved final consensus on a 25-item Safety Risk Assessment Checklist for Personalized Exercise. The checklist is organized into four core domains critical for a holistic, supportive care assessment:

#### Domain 1: medical and treatment-related factors (8 items)

Captures acute physiological safety parameters (e.g., neutropenia, fever, timing post-chemotherapy infusion).

#### Domain 2: symptom burden (5 items)

Assesses the severity of common treatment-related symptoms that directly impact tolerance and quality of life (e.g., severe fatigue, moderate-to-severe pain, active nausea).

#### Domain 3: functional and mobility considerations (5 items)

Evaluates physical impairments that increase injury risk and affect independence (e.g., neuropathy affecting balance, recent falls, symptomatic lymphedema).

#### Domain 4: patient-specific context (7 items)

Addresses psychosocial, comorbid, and behavioral factors influencing safe participation (e.g., unstable comorbidities, extreme sedentarism, high exercise-related anxiety).

The checklist is designed for binary (Yes/No) completion based on a current patient assessment, facilitating integration into routine nursing and supportive care clinical encounters. The checklist is intended for point-of-care use prior to each exercise session or at each relevant clinical encounter (e.g., pre-chemotherapy nursing assessment). This ‘session-by-session’ approach reflects the dynamic and fluctuating nature of risks during chemotherapy, where parameters such as blood counts and symptom severity can change rapidly [10]. The complete checklist is provided in Supplementary File 3 (Part A).

### Risk stratification and clinical decision guide

**Fig. 4:**
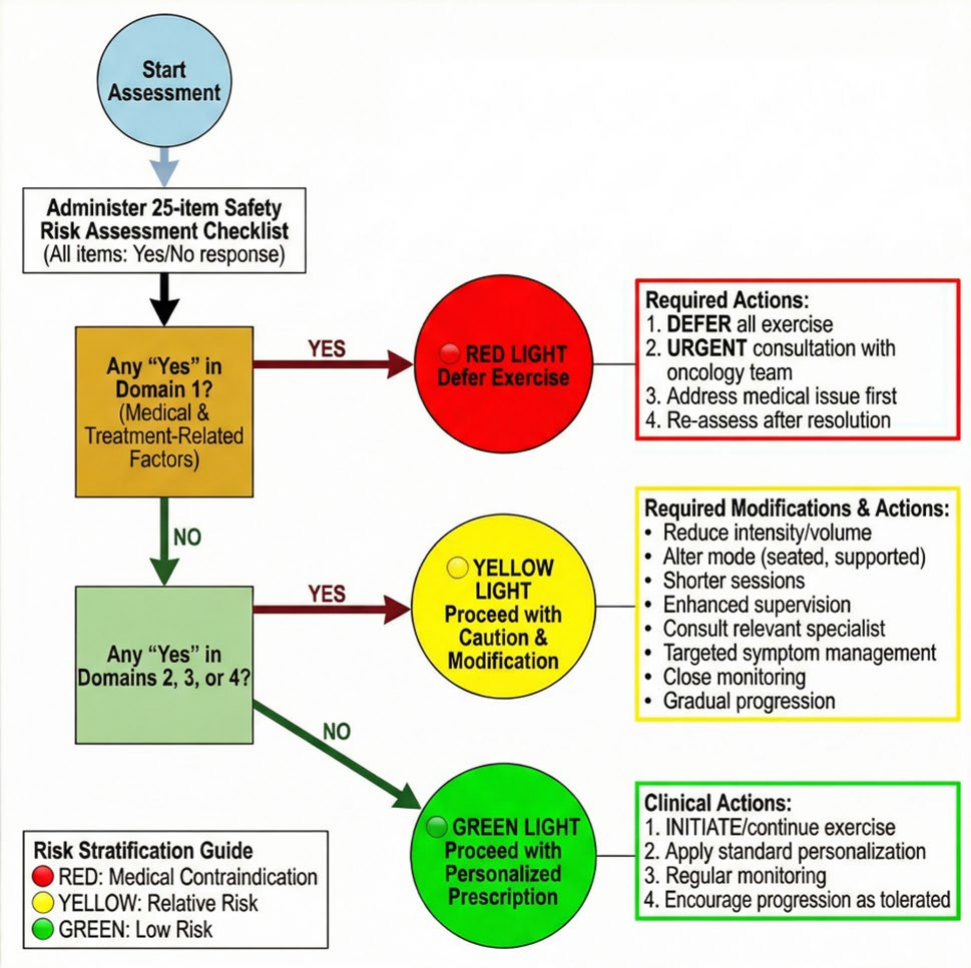
Clinical decision pathway based on the safety risk assessment checklist results

To translate assessment into actionable supportive care, a Risk Stratification Guide was developed. The three-tiered “traffic light” decision pathway was not included in the initial Round 1 checklist. During Round 2, several panelists noted that while the checklist identified risks, it did not provide clear guidance on how to act on those risks. Based on this qualitative feedback, the research team developed the Red-Yellow-Green stratification logic, which was then presented to the panel for informal feedback and refined iteratively. The final version achieved unanimous endorsement from all 20 panelists. It features a three-tiered decision pathway (Fig. 4) detailed in Supplementary File 3 (Part B):

#### Red light (defer and consult)

Triggered by any “Yes” in Domain 1. This signals an acute medical issue requiring resolution before exercise, prompting consultation with the oncology or supportive care team.

#### Yellow light (proceed with modifications)

Triggered by any “Yes” in Domains 2, 3, or 4 (provided Domain 1 is clear). This guides clinicians to tailor the exercise plan (e.g., modify intensity, mode) and consider referrals (e.g., to physical therapy for symptom management), enabling symptom-contingent activity. For instance, a “Yellow Light” triggered by “moderate fatigue” would guide the nurse to recommend starting with very low-intensity, short-duration activity (e.g., 10-minute slow walk) with close monitoring, rather than postponing exercise altogether.

#### Green light (proceed with personalized prescription)

Assigned when no items are checked “Yes,” supporting the initiation or continuation of appropriately prescribed exercise.

## Discussion

This study successfully developed and achieved robust multidisciplinary expert consensus on a 25-item Safety Risk Assessment Checklist and corresponding clinical decision pathway. The high participation rate and strong consensus across all Delphi rounds underscore the perceived clinical necessity of a standardized tool to facilitate the safe integration of exercise into supportive care for patients undergoing chemotherapy, thereby supporting its content validity [22, 23]. The final four-domain structure provides a comprehensive framework for a holistic supportive care assessment, ensuring critical dimensions—from acute medical status to symptom burden, functional integrity, and psychosocial context—are systematically evaluated to inform personalized activity planning [24, 25].

A pivotal feature of this checklist is its domain-specific risk stratification logic. Designating any “Yes” in Domain 1 (Medical & Treatment-Related Factors) as a “Red Light” prioritizes immediate medical attention for acute, unstable conditions, affirming the principle that patient safety and medical stabilization are paramount in supportive care [8, 26]. This is especially critical given the dynamic risk profile during chemotherapy, where factors like hematological status and symptom intensity fluctuate [10, 27]. Conversely, categorizing risks identified in Domains 2, 3, or 4 as a “Yellow Light” embodies a facilitative, symptom-contingent approach central to modern supportive care. It moves beyond binary exclusion towards tailored adaptation (e.g., modifying exercise intensity, mode, or environment) and underscores the importance of concurrent symptom management or rehabilitation referrals. This aligns with evidence that appropriately adapted physical activity is safe and beneficial even amidst manageable treatment-related challenges [28, 29]. The nuanced inclusion of items such as “current, symptomatic upper extremity lymphedema”—refined through expert deliberation to focus on active issues—explicitly aims to promote safe participation rather than impose blanket restrictions, consistent with best practice [6].

### Implications for supportive and nursing practice

This pragmatic tool offers a direct means to operationalize exercise within symptom management and supportive care plans. For oncology and palliative care nurses, who are frontline assessors of patient status, it can:

#### Standardize safety evaluation

Provide a consistent, evidence-informed framework for pre-activity assessment during routine clinical encounters (e.g., pre-chemotherapy reviews), reducing uncertainty and enhancing clinical decision-making [14].

#### Enhance patient-clinician communication and shared decision-making

The intuitive “traffic light” system offers a clear, visual tool for nurses to discuss individual risks, benefits, and adaptations with patients and families, directly addressing fears and promoting engagement in self-management strategies [30].

#### Guide interdisciplinary supportive care coordination

By offering clear triggers for consultation (“Red Light”) or referral (“Yellow Light” for specific symptoms/functional issues), the checklist facilitates structured communication and collaboration within the broader supportive care team, including physicians, physical therapists, and psychologists [25].

The checklist is designed with clear role allocation to support multidisciplinary workflow:

#### Nurses (primary intended users)

Complete the checklist during routine clinical encounters (e.g., pre-chemotherapy assessment). For ‘Green Light,’ proceed with general physical activity guidance. For ‘Yellow Light,’ implement symptom-contingent modifications within nursing scope. For ‘Red Light,’ defer exercise and consult the oncology team.

#### Physicians

Responsible for resolving ‘Red Light’ medical issues and providing clearance. May also use the checklist during medical assessments.

#### Physiotherapists/exercise physiologists

For ‘Yellow Light’ cases requiring specialized exercise prescription (e.g., neuropathy, balance issues), referral to rehabilitation specialists is recommended for individualized program design.

This multidisciplinary workflow is summarized in Table 1.

**Table 1:** Multidisciplinary roles in checklist implementation

Role	Primary Responsibilities	Trigger for Action	Clinical Examples
Nurse (primary intended user)	• Complete checklist during routine clinical encounters (e.g., pre-chemotherapy assessment) • For Green Light: provide general physical activity guidance and encouragement • For Yellow Light: implement symptom-contingent modifications within nursing scope (e.g., recommend low-intensity activity, monitor symptoms) • For Red Light: defer exercise and consult oncology team	All patients prior to exercise discussion or session	• Yellow Light (fatigue): recommend 10-min slow walk with rest breaks • Red Light (fever): defer exercise, notify physician
Physician	• Resolve Red Light medical issues • Provide medical clearance for exercise • May use checklist during medical assessments to inform treatment decisions	Red Light triggered; or when medical clearance needed	• Evaluate neutropenic patient, order repeat blood count • Clear patient to resume exercise after resolution of acute issue
Physiotherapist / Exercise Physiologist	• For Yellow Light cases requiring specialized prescription • Develop individualized exercise programs addressing specific impairments • Provide ongoing supervision and progression	Yellow Light triggered in Domains 2–3 (symptoms or functional limitations)	• Neuropathy affecting balance: prescribe proprioceptive training • Lymphedema: design gradual upper extremity strengthening program

Detailed individual expert profiles are provided in Supplementary File 2, Table S2.1

### Limitations and future directions

While this study establishes strong expert consensus, several limitations must be acknowledged, highlighting important avenues for future research within the supportive care context.

#### Scope and generalizability to palliative populations

The panel, though multidisciplinary, was composed of experts primarily from academic centers internationally (China, Europe, North America). The checklist was developed with a focus on patients receiving curative-intent chemotherapy. A significant limitation is that the checklist was developed specifically for breast cancer patients undergoing curative-intent chemotherapy, and the expert panel did not include palliative care specialists. Consequently, the tool’s applicability to patients with advanced or metastatic disease—where risk-benefit considerations differ substantially—remains unknown and should not be assumed. We recognize that the current tool targets early supportive care during active treatment rather than late palliative care. However, emerging evidence demonstrates that appropriately adapted exercise is safe, feasible, and beneficial even in the palliative phase for advanced cancer [31–33]. Therefore, future research must deliberately adapt and validate this checklist for advanced disease populations, with explicit involvement of palliative care specialists and patients with advanced disease from the outset.

#### Stage of validation and missing perspectives

This study establishes content validity. Additionally, the absence of pilot implementation data limits conclusions about the tool’s optimal frequency, feasibility, and clinical utility. The present study establishes content validity—the essential first step in tool development. Following the MRC framework for complex interventions [34], the next phases will involve: (1) a pilot feasibility study to establish administration protocols, assess acceptability, and refine items; (2) a prospective implementation study to evaluate impact on clinical decision-making and patient outcomes. Furthermore, the Delphi process did not include formal representation from patients, caregivers, or dedicated palliative care physicians. Future work must incorporate these vital stakeholder perspectives to ensure the tool’s acceptability, relevance, and alignment with patient-centered goals.

#### Operationalization of subjective symptoms

The checklist currently relies on clinical judgment for symptom assessment (e.g., ‘severe fatigue,’ ‘moderate-to-severe pain’), without providing standardized numerical cutoffs. This was an intentional decision by the expert panel to preserve clinical flexibility, as the functional impact of symptoms varies across individuals. However, this approach may limit inter-rater reliability and clinical operability. Future validation studies should explore integration with validated patient-reported outcome measures (e.g., PROMIS, Brief Fatigue Inventory) to enhance standardization while retaining the tool’s pragmatic nature [35].

#### Implementation considerations

The current format is a standalone checklist. Future research should explore its integration into electronic health records or patient-reported outcome systems to enhance usability and data capture [36]. Its potential to assess broader psychosocial barriers to activity engagement (e.g., illness-related stigma [37]) also warrants exploration. Furthermore, to support continuity of care, research is needed to examine how this ‘active treatment’ checklist can be transitioned or integrated with existing tools designed for the post-treatment survivorship phase, ensuring a seamless approach to long-term health promotion.

## Conclusion

This consensus study developed a pragmatic, oncology-specific safety assessment checklist and decision pathway. By providing a structured “Red-Yellow-Green” framework, it equips healthcare professionals, particularly nurses within the supportive care team, to more confidently and systematically integrate personalized exercise into the management of breast cancer patients during chemotherapy. The tool directly addresses a key implementation gap, translating guideline recommendations into actionable clinical practice to support symptom management and quality of life. The present study establishes content validity—the essential first step in tool development. Prospective studies to validate its utility, establish optimal administration protocols, and explore its adaptation across the cancer care continuum—including for patients with advanced disease with explicit involvement of palliative care specialists—are now essential.

## Supplementary Information

Supplementary Material 1.

Supplementary Material 2.

Supplementary Material 3.

Supplementary Material 4.

## Data Availability

The de-identified datasets generated and analyzed during the Delphi process are available from the corresponding author, Ying Song (songy1@jzmu.edu.cn), on reasonable request.

## Abbreviations

ANC: Absolute Neutrophil Count
IQR: Interquartile Range
QoL: Quality of Life
SD: Standard Deviation
