# Supplementary material for: A safety risk assessment checklist for personalized exercise as early supportive care in breast cancer patients undergoing chemotherapy: a modified Delphi consensus study
Source: BMC Palliat Care. 2026 Apr 1;25:137. doi: 10.1186/s12904-026-02083-3 (PMC13169593; doi:10.1186/s12904-026-02083-3)
Supplement: Supplementary file 1 — Supplementary Material 1. [file 12904_2026_2083_MOESM1_ESM.docx]

****Supplementary File 1: Complete Questionnaires for the Modified Delphi Consensus Process****

**File Description:**

**This file contains the full English-language survey instruments administered during the three-round modified online Delphi process, as well as the complete search strategies and data extraction form used in the scoping review that informed item generation. The questionnaires were delivered via the Qualtrics XM platform. They document the precise wording of items rated by the expert panel and the evolution of items based on feedback, ensuring methodological transparency and reproducibility for the study entitled "A Safety Risk Assessment Checklist for Personalized Exercise as Supportive Care in Breast Cancer Patients Undergoing Chemotherapy: A Modified Delphi Consensus Study."**

****1. Full Search Strategies****

**PubMed Search String (conducted March 2025)**

**("breast neoplasms"[MeSH Terms] OR "breast cancer"[Title/Abstract] OR "breast carcinoma"[Title/Abstract]) AND**

**("drug therapy"[MeSH Terms] OR "chemotherapy"[Title/Abstract] OR "antineoplastic agents"[MeSH Terms]) AND**

**("exercise"[MeSH Terms] OR "physical activity"[Title/Abstract] OR "resistance training"[Title/Abstract] OR "aerobic exercise"[Title/Abstract]) AND**

**("safety"[MeSH Terms] OR "adverse events"[Title/Abstract] OR "contraindications"[Title/Abstract] OR "risk assessment"[MeSH Terms]) AND**

**("symptoms"[Title/Abstract] OR "fatigue"[MeSH Terms] OR "neuropathy"[Title/Abstract] OR "pain"[MeSH Terms])**

**Filters: Publication date 2012-2025; English language**

****CINAHL Search String (conducted March 2025)****

**(MH "Breast Neoplasms+" OR TI "breast cancer" OR AB "breast carcinoma") AND**

**(MH "Antineoplastic Agents+" OR TI "chemotherapy" OR AB "drug therapy") AND**

**(MH "Exercise+" OR TI "physical activity" OR AB "resistance training") AND**

**(MH "Safety+" OR TI "adverse event*" OR AB "risk assessment") AND**

**(MH "Fatigue+" OR MH "Pain+" OR TI "neuropathy")**

**Filters: Publication date 2012-2025; English language; Peer reviewed**

****2. Data Extraction Form (Standardized Template)****

| **Field** | **Description** |
| --- | --- |
| Source ID | [Unique identifier] |
| Author(s) / Year | [Last name, year] |
| Source Type | □ Clinical practice guideline □ Screening tool □ Empirical study □ Other: _____ |
| Population | [Cancer type, treatment phase, sample size if applicable] |
| Safety Factor(s) Identified | [List specific safety considerations, contraindications, or risk factors] |
| Relevant Recommendation(s) | [Direct quotes or summary of guidance related to exercise safety] |
| Notes / Context | [Any additional context, e.g., evidence level, study limitations] |
| Extracted by | [Initials] |
| Date | [YYYY-MM-DD] |

****3. Delphi Survey — ROUND 1****

****Introduction to Panelists:****

**Thank you for participating in this Delphi study. The goal of this round is to evaluate the preliminary draft of a safety risk assessment checklist for prescribing exercise to breast cancer patients undergoing chemotherapy. Below is a list of items generated from a structured scoping review of guidelines and evidence. For each item, please rate its RELEVANCE (i.e., how important it is for assessing safety before initiating exercise) and its CLARITY (i.e., how clearly and unambiguously it is stated). Your open-ended feedback is crucial for refining the tool.**

**Instructions:**

**Relevance: Using the 5-point scale, indicate how relevant you believe the item is for determining if exercise is safe.**

**1 = Strongly Disagree (Not Relevant)**

**2 = Disagree**

**3 = Neither Agree nor Disagree**

**4 = Agree (Relevant)**

**5 = Strongly Agree (Highly Relevant)**

**Clarity: Using the 5-point scale, indicate how clearly the item is phrased for clinical use.**

**1 = Strongly Disagree (Not Clear)**

**2 = Disagree**

**3 = Neither Agree nor Disagree**

**4 = Agree (Clear)**

**5 = Strongly Agree (Very Clear)**

**Comments: For any item, please use the text box to suggest specific wording changes, propose merging with another item, or argue for its removal.**

**Preliminary Checklist Items for Rating (31 items):**

****Domain: Medical & Treatment Status****

**M1. The patient is within 48 hours before or after a chemotherapy infusion.**

**Relevance: 1 2 3 4 5 | Clarity: 1 2 3 4 5 | Comments: _________**

**M2. The patient has a fever (>38.0°C) or signs of active infection.**

**Relevance: 1 2 3 4 5 | Clarity: 1 2 3 4 5 | Comments: _________**

**M3. The patient's absolute neutrophil count (ANC) is < 0.5 × 10⁹/L.**

**Relevance: 1 2 3 4 5 | Clarity: 1 2 3 4 5 | Comments: _________**

**M4. The patient's platelet count is < 50 × 10⁹/L.**

**Relevance: 1 2 3 4 5 | Clarity: 1 2 3 4 5 | Comments: _________**

**M5. The patient's hemoglobin is < 80 g/L.**

**Relevance: 1 2 3 4 5 | Clarity: 1 2 3 4 5 | Comments: _________**

**M6. The patient has known or unstable cardiovascular disease.**

**Relevance: 1 2 3 4 5 | Clarity: 1 2 3 4 5 | Comments: _________**

**M7. There is evidence of acute or uncontrolled cardiotoxicity.**

**Relevance: 1 2 3 4 5 | Clarity: 1 2 3 4 5 | Comments: _________**

**M8. The patient reports new or worsening shortness of breath at rest.**

**Relevance: 1 2 3 4 5 | Clarity: 1 2 3 4 5 | Comments: _________**

****Domain: Symptom Burden****

**S1. The patient experiences severe fatigue (≥7 on a 0-10 scale) that limits daily activity.**

**Relevance: 1 2 3 4 5 | Clarity: 1 2 3 4 5 | Comments: _________**

**S2. The patient experiences moderate-to-severe pain (≥5 on a 0-10 scale) that is aggravated by movement.**

**Relevance: 1 2 3 4 5 | Clarity: 1 2 3 4 5 | Comments: _________**

**S3. The patient is experiencing active nausea/vomiting or diarrhea.**

**Relevance: 1 2 3 4 5 | Clarity: 1 2 3 4 5 | Comments: _________**

**S4. The patient reports significant dizziness or lightheadedness (presyncope).**

**Relevance: 1 2 3 4 5 | Clarity: 1 2 3 4 5 | Comments: _________**

**S5. The patient has unresolved severe post-operative complications.**

**Relevance: 1 2 3 4 5 | Clarity: 1 2 3 4 5 | Comments: _________**

****Domain: Functional & Mobility Limitations****

**F1. The patient has moderate-to-severe chemotherapy-induced peripheral neuropathy affecting balance or grip.**

**Relevance: 1 2 3 4 5 | Clarity: 1 2 3 4 5 | Comments: _________**

**F2. The patient has a history of breast cancer-related lymphedema.**

**Relevance: 1 2 3 4 5 | Clarity: 1 2 3 4 5 | Comments: _________**

**F3. The patient has musculoskeletal issues causing functional limitation.**

**Relevance: 1 2 3 4 5 | Clarity: 1 2 3 4 5 | Comments: _________**

**F4. The patient demonstrates impaired balance or has a history of recent falls (within the past month).**

**Relevance: 1 2 3 4 5 | Clarity: 1 2 3 4 5 | Comments: _________**

**F5. The patient has a central venous access device (e.g., port, PICC).**

**Relevance: 1 2 3 4 5 | Clarity: 1 2 3 4 5 | Comments: _________**

****Domain: Patient Context & Readiness****

**P1. The patient has significant, uncontrolled comorbidities (e.g., unstable angina, severe COPD).**

**Relevance: 1 2 3 4 5 | Clarity: 1 2 3 4 5 | Comments: _________**

**P2. The patient is completely sedentary and deconditioned.**

**Relevance: 1 2 3 4 5 | Clarity: 1 2 3 4 5 | Comments: _________**

**P3. The patient lacks social support or a safe environment for exercise.**

**Relevance: 1 2 3 4 5 | Clarity: 1 2 3 4 5 | Comments: _________**

**P4. The patient has a cognitive or psychological issue limiting comprehension or adherence.**

**Relevance: 1 2 3 4 5 | Clarity: 1 2 3 4 5 | Comments: _________**

**P5. The patient is receiving concurrent radiotherapy.**

**Relevance: 1 2 3 4 5 | Clarity: 1 2 3 4 5 | Comments: _________**

**P6. The patient has a high body mass index (BMI > 35 kg/m²).**

**Relevance: 1 2 3 4 5 | Clarity: 1 2 3 4 5 | Comments: _________**

**P7. The patient has a history of osteoporotic fracture.**

**Relevance: 1 2 3 4 5 | Clarity: 1 2 3 4 5 | Comments: _________**

**Open-Ended Section for New Item Suggestions:**

**Please suggest any additional safety factors not listed above that should be considered before prescribing exercise to this patient population.**

1. ****Delphi Survey — ROUND 2****

**Introduction to Panelists:**

**Thank you for your continued participation. This round presents a revised checklist based on your quantitative ratings and qualitative feedback from Round 1. For each item, please rate its RELEVANCE again. You will see the group's median relevance score from Round 1 and your own previous rating to inform your judgment.**

**Instructions:**

**Relevance Rating: Please re-rate each item's relevance using the same 5-point scale.**

**New Items: New items suggested in Round 1 have been integrated. Please rate their relevance.**

**Revised Checklist Items for Rating (26 items):**

**For each item, the survey displayed: [Item Number and Text] | [Median from R1] | [Your R1 Rating] | [Relevance Rating for R2: 1 2 3 4 5]**

****Domain 1: Medical & Treatment-Related Factors****

**D1.1 Within 48 hours before or after a chemotherapy infusion? | Med R1: 5.0 | Your R1: [Shown] | Rel R2: 1 2 3 4 5**

**D1.2 Current fever (>38.0°C) or signs of active infection? | Med R1: 5.0 | Your R1: [Shown] | Rel R2: 1 2 3 4 5**

**D1.3 Absolute neutrophil count (ANC) < 0.5 x 10⁹/L? | Med R1: 5.0 | Your R1: [Shown] | Rel R2: 1 2 3 4 5**

**D1.4 Platelet count < 50 x 10⁹/L? | Med R1: 4.5 | Your R1: [Shown] | Rel R2: 1 2 3 4 5**

**D1.5 Hemoglobin < 80 g/L? | Med R1: 4.0 | Your R1: [Shown] | Rel R2: 1 2 3 4 5**

**D1.6 Known, unstable, or suspected cardiovascular disease OR evidence of acute/uncontrolled cardiotoxicity? (Merged from M6 & M7) | Med R1: N/A | Your R1: N/A | Rel R2: 1 2 3 4 5**

**D1.7 New or worsening shortness of breath at rest? | Med R1: 4.0 | Your R1: [Shown] | Rel R2: 1 2 3 4 5**

****Domain 2: Symptom Burden****

**D2.1 Severe fatigue (≥7/10) limiting daily activities? | Med R1: 5.0 | Your R1: [Shown] | Rel R2: 1 2 3 4 5**

**D2.2 Moderate-to-severe pain (≥5/10) aggravated by movement? | Med R1: 5.0 | Your R1: [Shown] | Rel R2: 1 2 3 4 5**

**D2.3 Active nausea/vomiting or diarrhea? | Med R1: 4.0 | Your R1: [Shown] | Rel R2: 1 2 3 4 5**

**D2.4 Significant dizziness or presyncope? | Med R1: 4.0 | Your R1: [Shown] | Rel R2: 1 2 3 4 5**

****Domain 3: Functional & Mobility Considerations****

**D3.1 Neuropathy affecting balance or safety? | Med R1: 5.0 | Your R1: [Shown] | Rel R2: 1 2 3 4 5**

**D3.2 Current, symptomatic upper extremity lymphedema? (Revised from F2) | Med R1: 3.5 | Your R1: [Shown] | Rel R2: 1 2 3 4 5**

**D3.3 Musculoskeletal issues causing functional limitation? | Med R1: 4.0 | Your R1: [Shown] | Rel R2: 1 2 3 4 5**

**D3.4 Impaired balance or history of recent falls (past month)? | Med R1: 5.0 | Your R1: [Shown] | Rel R2: 1 2 3 4 5**

**D3.5 Issues with central venous access device site integrity or discomfort? (Revised from F5) | Med R1: 3.0 | Your R1: [Shown] | Rel R2: 1 2 3 4 5**

**Domain 4: Patient-Specific Context**

**D4.1 Uncontrolled comorbidities? | Med R1: 4.0 | Your R1: [Shown] | Rel R2: 1 2 3 4 5**

**D4.2 Extreme sedentarism/deconditioning? | Med R1: 4.0 | Your R1: [Shown] | Rel R2: 1 2 3 4 5**

**D4.3 High levels of exercise-related fear or anxiety? (New item) | Med R1: N/A | Your R1: N/A | Rel R2: 1 2 3 4 5**

**D4.4 Lack of social support or safe exercise environment? | Med R1: 4.0 | Your R1: [Shown] | Rel R2: 1 2 3 4 5**

**D4.5 Cognitive/psychological issue limiting comprehension? | Med R1: 4.0 | Your R1: [Shown] | Rel R2: 1 2 3 4 5**

**D4.6 Concurrent radiotherapy? (Item P5 from R1) | Med R1: 3.0 | Your R1: [Shown] | Rel R2: 1 2 3 4 5**

**D4.7 High body mass index (BMI > 35)? (Item P6 from R1) | Med R1: 2.5 | Your R1: [Shown] | Rel R2: 1 2 3 4 5**

****5. Delphi Survey — ROUND 3****

****Introduction to Panelists:****

**This final round presents the two items that did not achieve consensus for inclusion in Round 2. The wording has been refined based on the panel's written comments. Please provide your final RELEVANCE rating for these items.**

**Instructions:**

**Please rate the relevance of the following two items. Their final inclusion in the checklist depends on the consensus achieved in this round.**

**Items for Final Rating:**

**Item D3.2 (Final Revision): Does the patient have current, symptomatic upper extremity lymphedema?**

**Rationale for revision (shown to panelists): Feedback emphasized the need to assess for active, symptomatic states that require exercise modification (e.g., compression, mode adjustment) rather than a historical diagnosis.**

**Final Relevance Rating: 1 2 3 4 5**

**Item D3.5 (Final Revision): Are there issues with central venous access device (CVAD) site integrity (e.g., redness, swelling, pain, drainage) or does the patient report CVAD-related discomfort during arm movement?**

**Rationale for revision (shown to panelists): Feedback clarified that a well-healed, asymptomatic CVAD is not a contraindication. The item should focus on actionable clinical signs of infection or compromise that warrant caution.**

**Final Relevance Rating: 1 2 3 4 5**
