# Supplementary material for: A safety risk assessment checklist for personalized exercise as early supportive care in breast cancer patients undergoing chemotherapy: a modified Delphi consensus study
Source: BMC Palliat Care. 2026 Apr 1;25:137. doi: 10.1186/s12904-026-02083-3 (PMC13169593; doi:10.1186/s12904-026-02083-3)
Supplement: Supplementary file 2 — Supplementary Material 2. [file 12904_2026_2083_MOESM2_ESM.docx]

### ****Supplementary File 2: Demographic and Professional Characteristics of the Delphi Expert Panel****

****File Description:****

**This file provides detailed demographic and professional information for the 20 multidisciplinary experts who participated in the three-round modified Delphi consensus process. The composition of the panel was purposively sampled to ensure representation from all key clinical and scientific disciplines involved in the supportive care and rehabilitation of breast cancer patients undergoing chemotherapy, in accordance with the study methodology described in the main manuscript (Section 2.3.1).**

#### ****Table S2.1. Detailed Profile of Delphi Expert Panelists (N=20)****

| **Expert ID** | **Profession** | **Institution Type** | **Geographic Region** | **Years of Experience** | **Primary Clinical/Research Focus (related to study)** |
| --- | --- | --- | --- | --- | --- |
| E01 | Clinical Oncologist | Academic Medical Center | North America | 18 | Breast cancer medical oncology, cardiotoxicity management |
| E02 | Clinical Oncologist | Comprehensive Cancer Center | Europe | 12 | Breast cancer chemotherapy, survivorship care planning |
| E03 | Clinical Oncologist | University Hospital | Asia | 15 | Breast cancer treatment optimization, supportive care integration |
| E04 | Clinical Oncologist | Community Hospital | North America | 10 | Breast cancer care coordination, patient education |
| E05 | Clinical Oncologist | Teaching Hospital | Europe | 22 | Breast cancer clinical trials, toxicity management |
| E06 | Oncology Nurse Specialist | Comprehensive Cancer Center | North America | 9 | Chemotherapy administration, symptom management, patient education |
| E07 | Oncology Nurse Specialist | Academic Medical Center | Europe | 14 | Supportive care, fatigue management, exercise counselling |
| E08 | Oncology Nurse Specialist | University Hospital | Asia | 11 | Patient navigation, treatment adherence, quality of life assessment |
| E09 | Oncology Nurse Specialist | Community Hospital | North America | 7 | Symptom assessment, care coordination, psychosocial support |
| E10 | Oncology Nurse Specialist | Comprehensive Cancer Center | Europe | 13 | Exercise oncology implementation, patient monitoring, safety assessment |
| E11 | Exercise Physiologist | Academic Institution | North America | 16 | Cancer rehabilitation, exercise prescription, physical function assessment |
| E12 | Exercise Physiologist | Comprehensive Cancer Center | Europe | 8 | Exercise interventions during chemotherapy, adherence strategies |
| E13 | Exercise Physiologist | Research Institute | Asia | 12 | Exercise oncology research, biomarker response to exercise |
| E14 | Exercise Physiologist | University Department | North America | 19 | Exercise safety, risk stratification, personalized programming |
| E15 | Exercise Physiologist | Rehabilitation Center | Europe | 10 | Cancer-specific exercise protocols, functional capacity testing |
| E16 | Physical Therapist | Cancer Rehabilitation Center | North America | 17 | Lymphedema management, functional mobility, balance training |
| E17 | Physical Therapist | University Hospital | Europe | 13 | Neuropathy management, musculoskeletal rehabilitation |
| E18 | Physical Therapist | Private Practice | Asia | 9 | Post-surgical rehab, pain management, exercise adaptation |
| E19 | Physical Therapist | Academic Medical Center | North America | 21 | Cancer rehabilitation research, functional assessment tools |
| E20 | Physical Therapist | Comprehensive Cancer Center | Europe | 14 | Supportive care rehabilitation, multidisciplinary collaboration |

#### ****Geographic Distribution of the Expert Panel****

| Geographic Region | Number of Experts |
| --- | --- |
| China (Asia) | 12 |
| Europe (Germany, UK, Netherlands) | 5 |
| North America (USA, Canada) | 3 |
| Total | 20 |

#### ****Affiliation with Leading Professional Organizations****

#### **The expert panel includes members from major national and international professional societies in oncology, nursing, rehabilitation, and exercise science, underscoring the panel's engagement with established standards and ensuring the consensus is grounded in contemporary clinical practice.**

#### ****International and National Organizations Represented:****

#### **American Society of Clinical Oncology (ASCO): 3 experts**

#### **Oncology Nursing Society (ONS): 3 experts**

#### **American College of Sports Medicine (ACSM): 4 experts**

#### **Chinese Society of Clinical Oncology (CSCO): 4 experts**

#### **Chinese Nursing Association (CNA) - Oncology Nursing Committee: 4 experts**

#### **Chinese Association of Rehabilitation Medicine (CARM) - Cancer Rehabilitation Committee: 3 experts**

#### **Chinese Society of Exercise Physiology (CSEP): 3 experts**

#### **Chinese Anti-Cancer Association (CACA): 3 experts**

#### **Multinational Association of Supportive Care in Cancer (MASCC): 2 experts**

#### **Note: The panel includes experts from multiple countries. Geographic distribution: China (n=12), Europe (Germany, UK, Netherlands; n=5), North America (USA, Canada; n=3). All panelists confirmed active clinical practice and/or research focused on breast cancer patients undergoing chemotherapy at the time of the study. Their affiliation with leading professional organizations supports the validity and contextual relevance of their contributions to a consensus on a practical, clinically-grounded safety assessment tool, as discussed in the main manuscript (Section 3.1 & 4).**
