# Supplementary material for: A safety risk assessment checklist for personalized exercise as early supportive care in breast cancer patients undergoing chemotherapy: a modified Delphi consensus study
Source: BMC Palliat Care. 2026 Apr 1;25:137. doi: 10.1186/s12904-026-02083-3 (PMC13169593; doi:10.1186/s12904-026-02083-3)
Supplement: Supplementary file 3 — Supplementary Material 3. [file 12904_2026_2083_MOESM3_ESM.docx]

****Supplementary File 3: Safety Risk Assessment Checklist for Personalized Exercise in Breast Cancer Patients Undergoing Chemotherapy – Full Clinical Implementation Version****

**File Description:
This is the complete, ready-to-use version of the Safety Risk Assessment Checklist and its accompanying clinical implementation guide, as developed and agreed upon through the modified Delphi consensus process described in the main manuscript. This document is designed for direct use by oncology nurses, physical therapists, exercise physiologists, and other clinicians involved in the supportive care of breast cancer patients during chemotherapy.**

****PART A: THE 25-ITEM SAFETY RISK ASSESSMENT CHECKLIST****

**Instructions for Administration:**

**Purpose: To standardize the safety evaluation before initiating or modifying any exercise program for a breast cancer patient undergoing chemotherapy.**

**Timing: The checklist is intended for point-of-care use prior to each exercise session or at each relevant clinical encounter (e.g., pre-chemotherapy nursing assessment). This ‘session-by-session’ approach reflects the dynamic and fluctuating nature of risks during chemotherapy. Practically, the checklist should be completed based on a current patient assessment (within the past 7 days), with optimal integration points including pre-chemotherapy nursing assessment, weekly symptom review, or prior to a rehabilitation session.**

**Procedure: Read each item to the patient or review the patient's chart and recent self-reports. For each item, check “Yes” or “No” based on the patient's current status.**

**Action: After completing all items, proceed to PART B: Risk Stratification Guide to determine the appropriate clinical action (Green, Yellow, or Red Light).Patient ID: _______________________ Date: //_______
Clinician Name/Role: _________________________________**

| **Domain & Item No.** | **Checklist Item (Current Status)** | **Response (Check One)** |
| --- | --- | --- |
| **DOMAIN 1: MEDICAL & TREATMENT-RELATED FACTORS** |  |  |
| 1.1 | Is the patient within 48 hours before or after a chemotherapy infusion? | ☐ Yes ☐ No |
| 1.2 | Does the patient have a current fever (>38.0°C) or signs of active infection? | ☐ Yes ☐ No |
| 1.3 | Is the patient’s absolute neutrophil count (ANC) < 0.5 x 10⁹/L? | ☐ Yes ☐ No |
| 1.4 | Is the patient’s platelet count < 50 x 10⁹/L? | ☐ Yes ☐ No |
| 1.5 | Is the patient’s hemoglobin < 80 g/L? | ☐ Yes ☐ No |
| 1.6 | Does the patient have known, unstable, or suspected cardiovascular disease OR evidence of acute/uncontrolled cardiotoxicity? | ☐ Yes ☐ No |
| 1.7 | Does the patient report new or worsening shortness of breath at rest? | ☐ Yes ☐ No |
| **DOMAIN 2: SYMPTOM BURDEN** |  |  |
| 2.1 | Is the patient experiencing severe fatigue (≥7 on a 0-10 scale) limiting daily activities? | ☐ Yes ☐ No |
| 2.2 | Is the patient experiencing moderate-to-severe pain (≥5 on a 0-10 scale) aggravated by movement? | ☐ Yes ☐ No |
| 2.3 | Is the patient experiencing active nausea/vomiting or diarrhea? | ☐ Yes ☐ No |
| 2.4 | Does the patient report significant dizziness or presyncope? | ☐ Yes ☐ No |
| **DOMAIN 3: FUNCTIONAL & MOBILITY CONSIDERATIONS** |  |  |
| 3.1 | Does the patient have neuropathy affecting balance or safety? | ☐ Yes ☐ No |
| 3.2 | Does the patient have current, symptomatic upper extremity lymphedema? | ☐ Yes ☐ No |
| 3.3 | Does the patient have musculoskeletal issues causing functional limitation? | ☐ Yes ☐ No |
| 3.4 | Does the patient demonstrate impaired balance or a history of recent falls (past month)? | ☐ Yes ☐ No |
| 3.5 | Are there issues with central venous access device site integrity (e.g., redness, swelling, pain) or discomfort? | ☐ Yes ☐ No |
| **DOMAIN 4: PATIENT-SPECIFIC CONTEXT** |  |  |
| 4.1 | Does the patient have significant, uncontrolled comorbidities (e.g., unstable angina, severe COPD)? | ☐ Yes ☐ No |
| 4.2 | Is the patient extremely sedentary/deconditioned? | ☐ Yes ☐ No |
| 4.3 | Does the patient express high levels of exercise-related fear or anxiety? | ☐ Yes ☐ No |
| 4.4 | Does the patient lack social support or a safe environment for exercise? | ☐ Yes ☐ No |
| 4.5 | Is there a cognitive/psychological issue limiting comprehension or safety judgement? | ☐ Yes ☐ No |

**Clinician’s Global Assessment & Signature:
☐ Based on the above, the patient is medically stable for exercise participation at this time.
☐ All identified precautions have been noted and will be addressed in the care plan.
Signature: _________________________**

****PART B: RISK STRATIFICATION & CLINICAL DECISION GUIDE****

**Use the results from Part A to follow this algorithm. The goal is safe facilitation, not exclusion.**

**
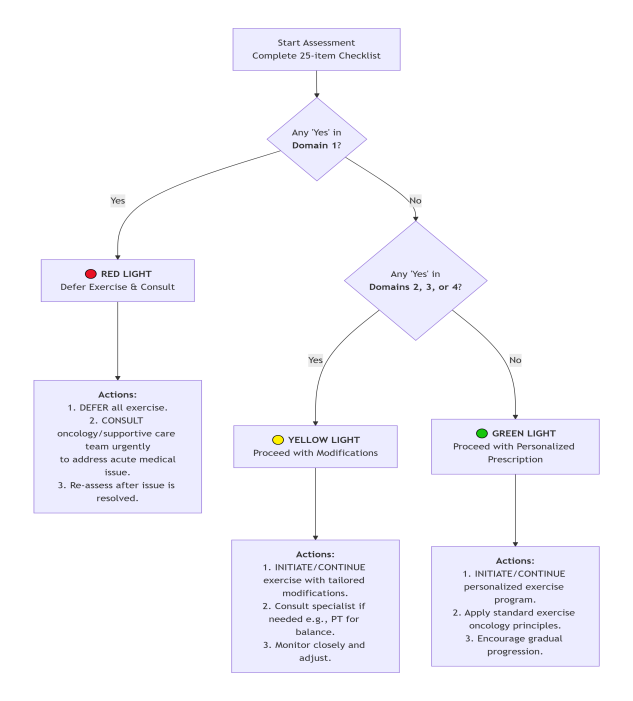
**

**Detailed Guidance for Each Pathway:**

**🔴 RED LIGHT (Defer & Consult)**

**Clinical Rationale: A “Yes” in Domain 1 indicates an acute, unstable medical or treatment-related condition where exercise could be harmful or interfere with urgent care. Safety is the absolute priority.**

**Required Actions:**

**Defer all exercise and document the specific trigger item(s).**

**Communicate promptly with the primary oncology team or relevant specialist (e.g., regarding fever, severe cytopenia, cardiac symptoms) to manage the acute issue.**

**Re-assessment is condition-based, not time-based. Re-administer the checklist only after the acute issue has been medically resolved and clearance is provided.**

**🟡 YELLOW LIGHT (Proceed with Modifications)**

**Clinical Rationale: A “Yes” in Domains 2, 3, or 4 identifies a manageable risk factor—a symptom, functional limitation, or contextual barrier. The goal is to adapt the exercise prescription to work around this factor safely, aligning with supportive care principles.**

**Required Actions & Modification Strategies:**

**Tailor the Exercise Plan: Modify the FITT principle (Frequency, Intensity, Time, Type).**

**For Symptoms (Domain 2): Reduce Intensity and Time (e.g., for severe fatigue: 10-min seated exercises, RPE <3/10). Choose a comfortable Type (e.g., recumbent cycling for pain).**

**For Functional Issues (Domain 3): Prioritize safety and support. Modify Type (seated exercises, avoid complex balance tasks for neuropathy). Ensure supervision.**

**For Contextual Factors (Domain 4): Address barriers. Start with familiar, low-skill activities to build confidence. Recommend supervised or home-based video programs.**

**Consider Referrals: Trigger referrals for concurrent management (e.g., to physical therapy for balance training, psychology for anxiety, dietetics for nutritional support).**

**Enhance Monitoring: Closely monitor the patient’s response during the first few sessions, particularly regarding the identified “Yellow Light” item.**

**🟢 GREEN LIGHT (Proceed with Personalized Prescription)**

**Clinical Rationale: The absence of red flags indicates the patient is medically stable for exercise. “Green” means proceed with a program personalized to the individual’s goals, treatment cycle, and baseline fitness, not a one-size-fits-all approach.**

**Required Actions:**

**Prescribe according to evidence-based guidelines (e.g., ACSM/ASCO), considering treatment timing (lower intensity near infusion days).**

**Set patient-centered goals and choose enjoyable activities to promote adherence.**

**Implement standard progression principles as tolerated.**

**PART C: DOCUMENTATION & MONITORING TEMPLATES**

**C.1. Initial Assessment and Decision Record**

**Patient ID: ________ Date: _______**

**Checklist Administrator (Role): _________________________**

**Risk Stratification Outcome: 🔴 Red / 🟡 Yellow / 🟢 Green**

**Key Trigger Items (if any): 1. __________ 2. __________**

**Clinical Decision & Plan:**

**(If Red): Exercise deferred. Consulted with Dr. ________ on _______. Issue: ________. Plan for re-assessment: ________.**

**(If Yellow): Modified exercise initiated. Key modifications: ________. Referral made to ________. Monitoring plan: ________.**

**(If Green): Standard personalized program initiated. Prescription: ________.**

**Patient Education Provided: ☐ Yes ☐ No. Topics covered: ________.**

**Clinician Signature: _________________________**

****C.2. Brief Session Monitoring Log (For Yellow/Green Light Patients)****

| **Date** | **Session Focus** | **Duration** | **Intensity (RPE 0-10)** | **Symptoms Pre/Post (e.g., Fatigue 0-10)** | **Notes/Adjustments** | **Initials** |
| --- | --- | --- | --- | --- | --- | --- |
|  |  |  |  |  |  |  |
|  |  |  |  |  |  |  |

**C.3. Recommended Re-assessment Triggers**

**Routine: Before each new chemotherapy cycle.**

**Event-driven: Upon any hospitalization, report of new/worsening symptoms (e.g., pain, falls, severe fatigue), or significant change in treatment plan.**
