# Supplementary material for: A safety risk assessment checklist for personalized exercise as early supportive care in breast cancer patients undergoing chemotherapy: a modified Delphi consensus study
Source: BMC Palliat Care. 2026 Apr 1;25:137. doi: 10.1186/s12904-026-02083-3 (PMC13169593; doi:10.1186/s12904-026-02083-3)
Supplement: Supplementary file 4 — Supplementary Material 4. [file 12904_2026_2083_MOESM4_ESM.docx]

### ****Supplementary Table S1: Delphi Process Statistics and Item Evolution****

****Table S1.**** Detailed results of the three-round modified Delphi consensus process. For each item, the median relevance score, interquartile range (IQR), and percentage agreement (percentage of panelists rating relevance as 4 or 5) are presented. The final column documents the disposition of each item based on consensus criteria (Consensus IN: Median ≥4.0, IQR ≤1.0, Agreement ≥80%).

| **Item ID** | **Provisional Domain** | **Original Wording (Round 1)** | **Round 1 Results (*n*=20)** | **Round 2 Wording & Results (*n*=20)** | **Round 3 Final Wording & Results (*n*=20)** | **Final Disposition & Notes** |
| --- | --- | --- | --- | --- | --- | --- |
|  |  |  | Med (IQR); % Agree | Med (IQR); % Agree | Med (IQR); % Agree |  |
| D1.1 | Medical | Within 48 hours pre/post chemotherapy | 5.0 (0); 100% | Retained, wording unchanged | N/A | Consensus IN. Key safety parameter. |
| D1.2 | Medical | Fever (>38°C) or active infection | 5.0 (0); 100% | Retained, wording unchanged | N/A | Consensus IN. Absolute contraindication. |
| D1.3 | Medical | ANC < 0.5 x 10⁹/L | 5.0 (0); 100% | Retained, wording unchanged | N/A | Consensus IN. Absolute contraindication. |
| D1.4 | Medical | Platelet count < 50 x 10⁹/L | 4.5 (1.0); 95% | Retained, wording unchanged | N/A | Consensus IN. |
| D1.5 | Medical | Hemoglobin < 80 g/L | 4.0 (1.0); 85% | Retained, wording unchanged | N/A | Consensus IN. |
| M6 | Medical | Known or unstable cardiovascular disease | 4.0 (1.0); 85% | Merged into D1.6 | N/A | Merged. Experts suggested combining overlapping cardiac risk items. |
| M7 | Medical | Evidence of acute/uncontrolled cardiotoxicity | 4.0 (1.0); 90% | Merged into D1.6 | N/A | Merged. |
| D1.6 | Medical | N/A | N/A | 5.0 (0); 100% (New merged item) | N/A | Consensus IN. Final item: "Known, unstable, or suspected cardiovascular disease OR evidence of acute/uncontrolled cardiotoxicity?" |
| D1.7 | Medical | New/worsening shortness of breath at rest | 4.0 (1.0); 85% | Retained, wording unchanged | N/A | Consensus IN. |
| D2.1 | Symptom | Severe fatigue (≥7/10) limiting activity | 5.0 (0); 100% | Retained, wording unchanged | N/A | Consensus IN. Core symptom for supportive care. |
| D2.2 | Symptom | Moderate-to-severe pain (≥5/10) aggravated by movement | 5.0 (0); 100% | Retained, wording unchanged | N/A | Consensus IN. Core symptom for supportive care. |
| D2.3 | Symptom | Active nausea/vomiting or diarrhea | 4.0 (1.0); 85% | Retained, wording unchanged | N/A | Consensus IN. |
| D2.4 | Symptom | Significant dizziness or presyncope | 4.0 (1.0); 85% | Retained, wording unchanged | N/A | Consensus IN. |
| S5 | Symptom | Unresolved severe post-op complications | 3.0 (2.0); 60% | Dropped | N/A | Consensus OUT. Deemed too rare/non-specific for this checklist; covered by other acute medical items. |
| D3.1 | Functional | Neuropathy affecting balance or safety | 5.0 (0); 100% | Retained, wording unchanged | N/A | Consensus IN. |
| F2 | Functional | History of lymphedema | 3.5 (2.0); 65% | Revised to "Current, symptomatic lymphedema" (Item D3.2) | See D3.2 | Revised & Carried to R3. Major debate on history vs. active state. |
| D3.2 | Functional | N/A | N/A | 3.5 (1.5); 70% (As revised item) | 4.0 (1.0); 90% | Consensus IN (after R3). Final item: "Current, symptomatic upper extremity lymphedema?" |
| D3.3 | Functional | Musculoskeletal issues causing functional limitation | 4.0 (1.0); 85% | Retained, wording unchanged | N/A | Consensus IN. |
| D3.4 | Functional | Impaired balance or recent falls | 5.0 (0); 100% | Retained, wording unchanged | N/A | Consensus IN. |
| F5 | Functional | Presence of a CVAD | 3.0 (2.0); 55% | Revised to "CVAD site issues/discomfort" (Item D3.5) | See D3.5 | Revised & Carried to R3. Consensus that presence alone is not a risk factor. |
| D3.5 | Functional | N/A | N/A | 4.0 (1.5); 75% (As revised item) | 4.0 (1.0); 85% | Consensus IN (after R3). Final item: "Issues with central venous access device site integrity or discomfort?" |
| D4.1 | Context | Uncontrolled comorbidities | 4.0 (1.0); 85% | Retained, wording unchanged | N/A | Consensus IN. |
| D4.2 | Context | Extreme sedentarism/deconditioning | 4.0 (1.0); 85% | Retained, wording unchanged | N/A | Consensus IN. |
| N/A | Context | N/A (Suggested in R1) | N/A | "High exercise-related fear/anxiety" (Item D4.3) | N/A | New Item, Consensus IN. Added based on panelist suggestion. Final item: "High levels of exercise-related fear or anxiety?" R2 Result: 4.0 (1.0); 95% |
| D4.4 | Context | Lack of social support/safe environment | 4.0 (1.0); 85% | Retained, wording unchanged | N/A | Consensus IN. |
| D4.5 | Context | Cognitive/psychological issue limiting comprehension | 4.0 (1.0); 85% | Retained, wording unchanged | N/A | Consensus IN. |
| P5 | Context | Concurrent radiotherapy | 3.0 (2.0); 60% | Included as Item D4.6 for R2 rating | N/A | Consensus OUT. R2 Result for D4.6: 2.5 (1.5); 40%. Deemed not a primary safety risk for exercise during chemotherapy; timing/field-specific concerns too variable. |
| P6 | Context | High BMI (>35) | 2.5 (2.0); 45% | Included as Item D4.7 for R2 rating | N/A | Consensus OUT. R2 Result for D4.7: 2.0 (2.0); 30%. Deemed not an independent risk factor for exercise safety in this context; approach should be based on functional assessment, not BMI alone. |
| P7 | Context | History of osteoporotic fracture | 2.0 (2.0); 40% | Dropped | N/A | Consensus OUT. |
| Summary |  | 31 Items Rated | 22 IN, 7 No Consensus, 2 New Suggested | 26 Items Rated (22 retained+2 merged+2 new) | 2 Items Rated | Final Consensus achieved on all 25 items. |
|  |  |  |  | 24 IN, 2 No Consensus | 2 IN, 0 Out | Items not reaching consensus in R2 (D3.2, D3.5) were revised and achieved consensus in R3. Items D4.6 and D4.7 did not reach consensus and were removed. |

****Abbreviations:**** ANC: Absolute Neutrophil Count; CVAD: Central Venous Access Device; BMI: Body Mass Index; Med: Median; IQR: Interquartile Range; % Agree: Percentage of ratings ≥4.
